# Core immune cell infiltration signatures identify molecular subtypes and promote precise checkpoint immunotherapy in cutaneous melanoma

**DOI:** 10.3389/fimmu.2022.914612

**Published:** 2022-08-22

**Authors:** Zheng Zhu, Guoyin Li, Zhenning Li, Yinghua Wu, Yan Yang, Mingyang Wang, Huihua Zhang, Hui Qu, Zewen Song, Yuanmin He

**Affiliations:** ^1^ Department of Medicine, Harvard Medical School, Boston, MA, United States; ^2^ Key Laboratory of Modern Teaching Technology, Ministry of Education, Shaanxi Normal University, Xi’an, China; ^3^ Department of Oromaxillofacial-Head and Neck Surgery, Liaoning Province Key Laboratory of Oral Disease, School and Hospital of Stomatology, China Medical University, Shenyang, China; ^4^ School of Medicine, Central South University, Changsha, China; ^5^ Department of Public Health, Southwest Medical University, Luzhou, China; ^6^ Department of Ophthalmology, Chinese People's Liberation Army (PLA) General Hospital, Beijing, China; ^7^ Department of Plastic Surgery, Shanxi Bethune Hospital, Shanxi Academy of Medical Sciences, Taiyuan, China; ^8^ Department of Oncology, The Third Xiangya Hospital of Central South University, Changsha, China; ^9^ Department of Dermatology, The Affiliated Hospital of Southwest Medical University, Luzhou, China

**Keywords:** cutaneous melanoma, tumor microenvironment, prognosis, immunotherapy, cTICscore

## Abstract

Yutao Wang, China Medical University, ChinaThe tumor microenvironment (TME) has been shown to impact the prognosis of tumors in patients including cutaneous melanoma (CM); however, not all components of TME are important. Given the aforementioned situation, the functional immune cell contents correlated with CM patient prognosis are needed to optimize present predictive models and reflect the overall situation of TME. We developed a novel risk score named core tumor-infiltrating immune cell score (cTICscore), which showed certain advantages over existing biomarkers or TME-related signatures in predicting the prognosis of CM patients. Furthermore, we explored a new gene signature named cTILscore−related module gene score (cTMGs), based on four identified TME-associated genes (*GCH1*, *GZMA*, *PSMB8*, and *PLAAT4*) showing a close correlation with the cTICscore, which was generated by weighted gene co-expression network analysis and least absolute shrinkage and selection operator analysis to facilitate clinical application. Patients with low cTMGs had significantly better overall survival (OS, *P* = 0.002,< 0.001, = 0.002, and = 0.03, respectively) in the training and validating CM datasets. In addition, the area under the curve values used to predict the immune response in four CM cohorts were 0.723, 0.723, 0.754, and 0.792, respectively, and that in one gastric cohort was 0.764. Therefore, the four-gene signature, based on cTICscore, might improve prognostic information, serving as a predictive tool for CM patients receiving immunotherapy.cutaneous melanoma, tumor microenvironment, prognosis, immunotherapy, cTICscore

## Introduction

Cutaneous melanoma (CM) is one of the most aggressive skin cancers, with 324,635 new cases and 57,043 deaths reported in 2020 worldwide (1). CM has long been considered an immunogenic cancer because of its highly mutagenized genome, making it one of the most responsive cancer types to immunotherapies (2). However, some patients showed unresponsiveness or acquired resistance using these immunotherapeutic approaches (3). Studies showed that the response rate to checkpoint blockade of PD-1 or programmed death-ligand 1 (PD-L1) signaling was around one-third (4). Another study showed that the effect of PD-1/PD-L1 therapy for patients correlated with PD-L1 expression in the tumor microenvironment (TME) (5).

Based on the difference in responsiveness, an increasing number of studies were conducted to work out models so as to identify better biomarkers for CM prognosis under immunotherapy. Zihang Zeng and colleagues developed a novel scoring system named ISTMEscore to reflect the TME status and predict CM prognosis (6). Dongqiang Zeng developed an open-source TMEscore serving as a promising predictive index (7). These studies were complete and creative while they took into account relatively insignificant genes, thus reducing the effectiveness of their model. Bagaev et al. identified four TME subtypes predictive of response for multiple cancers in terms of immunotherapy (8). However, a large number of genes and their expression levels were examined, and no significant difference in the overall survival (OS) between immune-enriched, non-fibrotic (IE) and immune-enriched, fibrotic (IE/F) subtypes was observed in the study in terms of the responsiveness to immunotherapies. TME comprises a variety of infiltrated immune cells and stromal cells such as endothelial cells and fibroblasts (9), in which abnormal cells finally become cancerous and accumulate (10). Extensive studies confirmed the significant role of the proportion of tumor-infiltrating immune cells in patient prognosis. For instance, tumor-associated macrophages indicated an unfavorable prognosis because of the immune events they triggered, such as secreting cytokine interleukin-10 (IL-10) (11). The increase in the number of CD4^+^ T cells and CD8^+^ T cells was associated with better response and survival (12). However, some studies ignored the comprehensive interactions among different types of immune cells while focusing only on specific ones, such as CD8^+^ T cells (13). Given the aforementioned situation, the functional immune cell contents correlated with patient prognosis are needed to optimize present predictive models and reflect the overall situation of TME. A more robust index is needed for more precise evaluation.

In the present study, we selected prognosis-related tumor-infiltrating immune cells and established a new index named core tumor-infiltrating immune cell score (cTICscore) for CM subtype identification, which was shown to be robust for characterizing TME and predicting CM patients’ prognosis. Further, a model involving four crucial genes and correlated with the cTICscore was generated for convenience in clinical application. The new model showed some superiority in predicting CM patients’ prognosis over existing signatures or biomarkers and could provide guidance for the choice of clinical tumor immunotherapies.

## Material and methods

### Data acquisition and processing

The downloading and processing of the data of CM patients from the Cancer Genome Atlas (TCGA_SKCM) database was conducted as reported in our previous study (14). Normalized gene expression data and clinical data of datasets from the Gene Expression Omnibus (GEO) database (GSE65904, GSE22153, GSE54467, GSE100797, GSE35640, and GSE176307) were acquired *via* the *GEOquery* package in R software or from the supplementary files of the corresponding publications (15–20). Processed RNA-seq data and clinical information for the Peking University Cancer Hospital (PUCH) study, Gide19 study, and Kim18 study were downloaded from the GitHub website (https://github.com/) as reported in the Chuanliang Cui’s study (21). The data of the IMvigor210 study were downloaded from the reported website (http://research-pub.gene.com/IMvigor210CoreBiologies/) in the study by Sanjeev Mariathasan (22). The single-cell RNA-seq data of selected genes in four GEO datasets, namely, GSE72058, GSE148190, GSE123139, and GSE115978, were downloaded from the TISCH website (http://tisch.comp-genomics.org/). The expression level of selected genes in immune cells and malignant tumor cells at the single-cell level in a melanoma sample from the study by Wu was directly visualized in and downloaded from the Single Cell Portal website (https://singlecell.broadinstitute.org/single_cell) (23). All data used in this study were acquired from public databases; further approval from an ethics committee was not required.

### Immune profile analysis

The infiltration level of 22 immune cells in each tumor sample was evaluated *via* the CIBERSORT algorithm in R software (24). The Immunescore of each tumor sample was estimated using the ESTIMATE algorithm in the *estimate* package in R software (25). The enrichment score of 29 functional gene expression signatures (Fges) in each CM sample was downloaded from the corresponding study (8).

### Enrichment analysis

The enrichment score of specific pathways was calculated using the GSVA package in R software (26). The used gene set *C2.cp.kegg.v7.1.symbols.gmt* was downloaded from the gene set enrichment analysis website (http://www.gsea-msigdb.org/gsea/index.jsp). The metabolic-related and other specific biological-related gene sets were acquired from corresponding publications (22, 27).

### Weighted gene co-expression network analysis

Weighted gene co-expression network analysis (WGCNA) was conducted in R software based on the instruction and R tutorial from Peter Langfelder et al. (22). Briefly, the gene expression data of each dataset, after removing genes and samples with too many missing values, were used to construct a gene co-expression network. An adjacency matrix was subsequently constructed to calculate the correlation strength between the nodes using the following formula:


$$s_{ij}=\;\left|cor\left(x_{i}\;,\;x_{j}\right)\right|a_{ij}=\;S_{ij}^{\beta }$$

The co-expression similarity *S*_ij_ represents the Pearson’s correlation coefficient between two different genes *i* and *j*. *X_i_* and *x_j_* are the corresponding expression values of the genes *i* and *j*, and *a_ij_* is the correlation strength between the two genes. The scale-free *R* ^2^ was set as 0.9 to select the corresponding soft-threshold β. One-step network construction and module detection methods were subsequently used, with a relatively large minimum module size of 200 and *mergeCutHeight* setting as 0.25 for the merging of modules. Finally, module–trait associations were quantified to identify modules significantly associated with the cTICscore and Immunescore. Besides, the definition and expression of module eigengenes (MEs), the gene significance (GS), and the module significance (MS) were similar with what had been described in a previous study (28).

### Construction of the prognostic model

The prognostic significance of the infiltration of 22 immune cells in CM was evaluated by univariate Cox analysis. Immune cells showing a *P* value less than 0.1 in all three datasets were further subjected to multivariate Cox analysis (29). A score (score1) was calculated by multiplying the coefficient of each immune cell and its infiltration level in each sample, namely, score1 = –3.13613 × Macrophages_M1 – 0.98753 × T_cells_CD8 – 2.4095 ×T_cells_CD4_memory_activated + 1.69976 × NK_cells_resting, and cTICscore = (score1-Min)/absolute (Max), as reported in our previous studies (30, 31). The selected cTICscore-related genes were input into the Least Absolute Shrinkage and Selection Operator (LASSO) Cox regression model, and crucial gene signatures were generated *via* the *glmnet* package in R. The corresponding coefficients of the generated crucial genes were obtained through multivariate Cox analysis. A second score (score2) was calculated as score2 = –0.08209 × PSMB8 – 0.02401 × PLAAT4 – 0.18873 × GZMA – 0.19433 × GCH1, and the cTICscore-related module gene signature (cTMGs) was also calculated using the following formula: cTMGs = (score2 – Min)/absolute (Max).

### Statistical analysis

The median value of cTICscore or cTMGs in each cohort was used as the cutoff value in separating patients into two subgroups. Univariate Cox regression analyses were conducted to determine the prognostic significance of the infiltrating level of 22 immune cells in melanoma datasets using the *survminer* package in R. The same package was also used for multivariate Cox regression in obtaining coefficients of the four core TME components or four crucial genes. The Kaplan–Meier method with the log-rank test was used for survival analyses. The *timeROC* package in R was applied for time-dependent receiver operator characteristic (ROC) analyses and subsequent calculation of the area under the curve (AUC). This work also took advantage of the following packages in R for data analyses and graph plotting: tidyverse, limma, ggplot2, rms, dplyr, plyr, ggpubr, ggalluvial and vennDiagram. *P*< 0.05 indicated statistically significant differences (^*^, *P*< 0.05; ^**^, *P*< 0.01; ^***^, *P*< 0.001; ^****^, *P*< 0.0001).

## Results

### Core tumor−infiltrating immune cell score (cTICscore) identified CM subtypes with distinct prognosis

**Figure 1:**
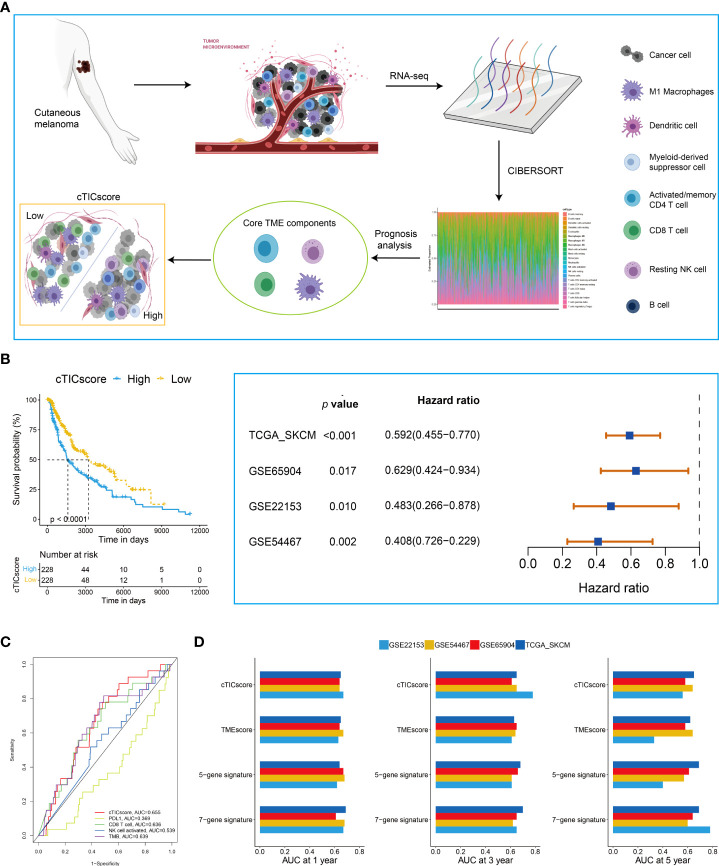
Identification of CM subtypes with distinct prognoses based on the cTICscore. **(A)** Illustration for the construction of the cTICscore in CM. **(B)** cTICscore-based CM subtypes exhibited distinct prognoses. **(C)** Time-dependent ROC curves for the cTICscore and other predictors. **(D)** AUC of the time-dependent ROC curves for the cTICscore, TMEscore, the five-gene signature and the seven-gene signature.

The flowchart to develop a cTICscore for CM patients is shown in **Figure 1A**. Briefly, the fraction of 22 immune cells was estimated using the CIBERSORT algorithm in three independent CM-related datasets, namely, TCGA-SKCM, GSE65094, and GSE22153. Univariate Cox analysis was used to evaluate the prognostic relevance of these immune cells, and core TME components referred to those that showed a *P* value less than 0.1 in all these three datasets (29). These components were identified to be activated memory CD4^+^T cells, CD8^+^T cells, resting natural killer (NK) cells, and macrophages M1 (**Figure 1A**, **Table S1**).

Next, we calculated the cTICscore of CM patients in each cohort. CM patients stratified into two subgroups using the median cTICscore as the cutoff value showed distinct prognosis in all the three datasets and the external validating GSE54467 (**Figure 1B**). Previous studies suggested that NK cell–related signature, CD8^+^T cell-related signature, PD-L1 expression, or TMB could be used to predict the prognosis of CM patients (14, 32, 33). The cTICscore, compared with these predictors, seemed to have better predictability based on the AUC of the time-dependent ROC curves (**Figure 1C**). Dongqiang Zeng et al. developed a package calculating the TMEscore of individual cancer samples to characterize the TME of gastric cancer (7, 34). Although the TMEscore can also help to stratify CM patients having a distinct prognosis (**Figure S1A**), the AUC value at 5 years of the TMEscore in the GSE22153 dataset was quite low (less than 0.5, **Figure 1D**), suggesting that the TMEscore was slightly inferior to the cTICscore in predicting the OS of melanoma patients. We also included two other immune-related signatures in this work; while the seven-gene signature from Tian’s study (35) had a similar predictability like the cTICscore, the five-gene signature from Hu’s study (36) also had a low AUC value at 5 years in the GSE22153 dataset (**Figure 1D**). Taken together, the developed cTICscore showed a certain advantage over the existing method in characterizing the TME and predicting the CM patients’ prognosis.

Kyoto Encyclopedia of Genes and Genomes (KEGG) enrichment analysis was performed to characterize further the features of the high- and low-cTICscore groups. We found that the low-cTICscore group was highly enriched in immune-related pathways such as antigen processing and presentation and NK cell–mediated cytotoxicity; however, the high-cTICscore group was enriched in metabolism-related pathways such as aminoacyl tRNA biosynthesis, glycosaminoglycan biosynthesis, and keratan sulfate, and lysine degradation (**Figure S1B**).

### Development of the cTMGs in CM patients

**Figure 2:**
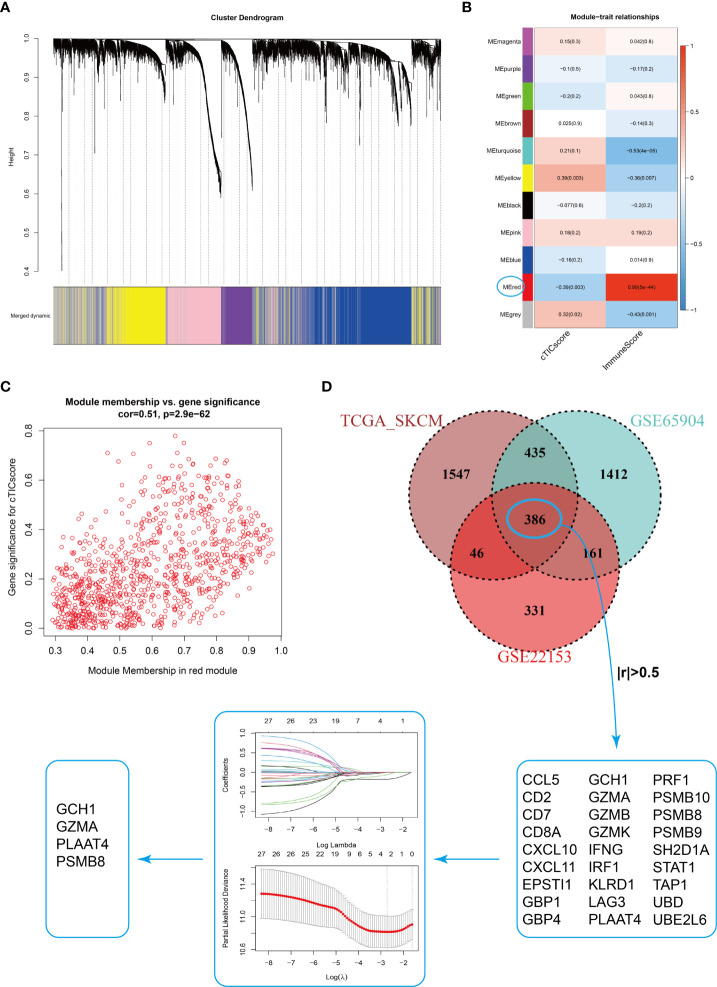
Identification of four signature genes in CM TME. **(A)** Merging of mRNA co-expression modules. **(B)** Correlation heatmap of module genes and cTICscore or Immunescore in the GSE22153 dataset. The correlation coefficient changed from –1 to 1 as the color turned from blue to red gradually. **(C)** Scatterplot of the correlation coefficient between the selected module (red module) and the cTICscore. **(D)** Flowchart of the identification of four signature genes in CM TME. A total of 386 genes were shared in the selected modules from TCGA_SKCM, GSE65904, and GSE22153, and 27 of these genes strongly correlated (absolute coefficient > 0.5) in expression with the cTICscore across all three datasets. The 27 genes were input into a LASSO regression model, which generated four crucial genes for further analysis.

Estimating the fraction of infiltrated immune cells in the TME using algorithms such as CIBERSORT depended on the availability of transcriptional data of thousands of genes and might be prone to different unexpected biases (37, 38). We hypothesized that a specific fingerprint, consisting of several genes reflecting the infiltration of core TME components of CM patients, might be more applicable for clinical purposes. WGCNA was first applied to identify modules highly correlated with the cTICscore (**Figure 2A**). The red module in GSE22153 (**Figure 2B**), the turquoise module in GSE65904 (**Figure S2A**), and the brown module in TCGA_SKCM (**Figure S2B**) were found to be most significantly negatively associated with the cTICscore but positively associated with the Immunescore calculated *via* the ESTIMATE algorithm. Besides, the red module in GSE22153 (cor = 0.51, *P* = 2.9e-62, **Figure 2C**), the turquoise module in GSE65904 (cor = 0.53, *P* = 1.9e-173, **Figure S2C**), and the brown module in TCGA_SKCM (cor = 0.77, *P*< 1e−200, **Figure S2D**) indicated a high GS in relation to the cTICscore. A total of 386 genes were shared among the red module of GSE22153, the turquoise module of GSE65904, and the brown module of TCGA_SKCM (**Figure 2D**). In addition, 27 of the 386 genes strongly correlated in transcriptional expression with the cTICscore in all the three datasets (**Figure 2D**). These 27 genes were further input into a LASSO regression model, which generated four crucial genes, including GTP cyclohydrolase 1 (*GCH1*), granzyme A (*GZMA*), proteasome subunit beta type-8 (*PSMB8*), and phospholipase A and acyltransferase 4 (*PLAAT4*) (**Figure 2D**).

### Characterization of the four crucial genes in CM TME

**Figure 3:**
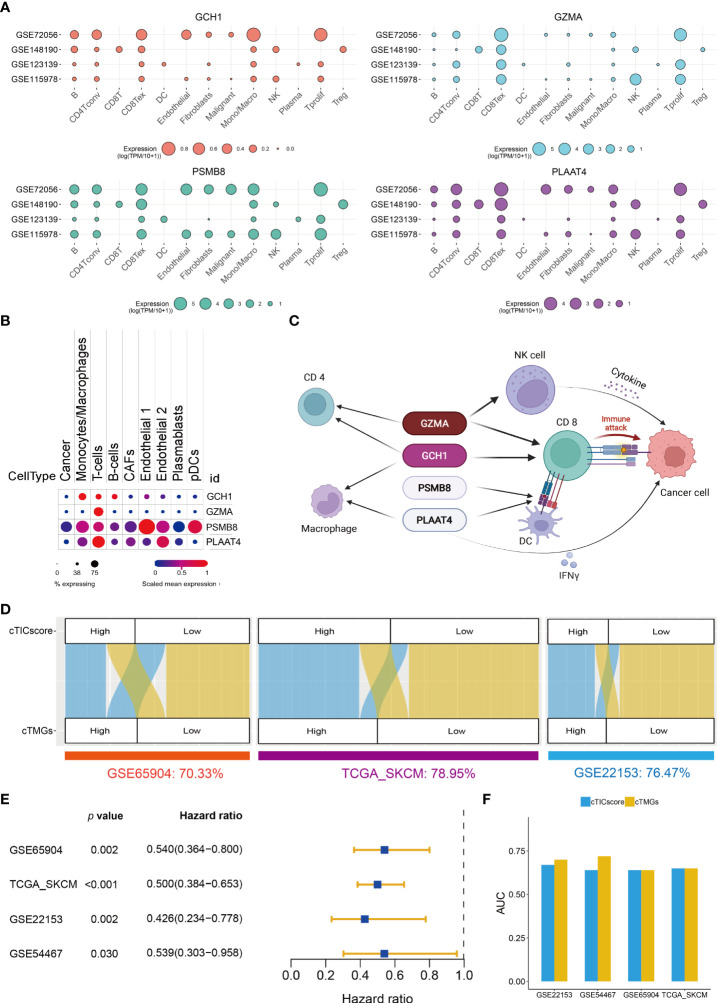
Construction of cTMGs based on four signature genes. **(A)** Expression of the four crucial genes in the TME components of CM. **(B)** Expression of the four crucial genes in different types of cells. **(C)** Schematic description of the potential functions of the four crucial genes in CM. **(D)** Relationship between cTMGs-based subtypes and cTICscore subtypes in different datasets. **(E)** Kaplan–Meier survival analysis results of patients in the high- and low-cTMGs groups. **(F)** AUC of the time-dependent ROC curves for the cTICscore and cTMGs.

The prognostic analysis showed that CM patients with the high expression of one of the four crucial genes, when stratified by the median value of its expression, had significantly longer OS (**Figures S3A–D**). GCH1 functions as the first and rate-limiting enzyme in tetrahydrobiopterin biosynthesis (BH4). A recent study revealed that GCH1 had a remarkably positive impact on T-cell proliferation and immune response in autoimmunity and cancer (39). GZMA is predominantly expressed in cytolytic T lymphocytes and NK cells and is necessary for the execution of lysis of target cells (40). PSMB8 is a major component of the immunoproteasome, which is found predominantly in monocytes and lymphocytes and known for processing class I major histocompatibility complex (MHC-I) (41). PLAAT4, also known as retinoic acid receptor responder 3 (RARRES3) or retinoid-inducible gene 1 (RIG-1), is characterized as a tumor suppressor and plays a role in the induction of type I interferon (IFN-1) and MHC-I expression (42, 43). The expression of all these four genes significantly correlated with the four core TME components of CM (**Figure S3E**). In particular, the transcriptional level of all the four genes had a strong positive correlation with the infiltration of CD8^+^ T cells (*R* > 0.5, **Figure S3E**). Besides, GCH1 and GZMA also strongly correlated with the fraction of the activated memory CD4^+^ T cells (*R* > 0.5, **Figure S3E**). A single-cell transcriptional analysis of four independent datasets indicated that GCH1 had a relatively high expression in monocytes/macrophages, B cells, CD4^+^ T cells, and CD8^+^ T cells, but low or no expression in malignant tumor cells (**Figure 3A**). GZMA was predominantly expressed in CD8^+^ T cells, proliferative T cells, and NK cells (**Figure 3A**). PSMB8 could be detected with a relatively high expression in various immune cells and tumor cells (**Figure 3A**), and PLAAT4 showed a relatively high expression in CD4^+^ T cells, CD8^+^ T cells, NK cells, and proliferative T cells (**Figure 3A**). The expression pattern of the four crucial genes was further supported by another study (23) (**Figure 3B**), which demonstrated that all four genes were highly expressed in T cells. In addition, PSMB8 could be detected in most cells, with a relatively high expression in monocytes/macrophages, T cells, endothelial cells, and plasmacytoid dendritic cells (pDCs) (**Figure 3B**). GCH1 was highly expressed in monocytes/macrophages, T cells, and B cells (**Figure 3B**). GZMA was predominantly expressed in most T cells, while PLAAT4 was predominantly expressed in T cells and endothelial cells (**Figure 3B**). Taken together, all four crucial genes had important roles in the TME and significantly correlated with the infiltration of four core TME components. Their potential function in CM is shown in **Figure 3C**.

These LASSO-selected genes were further used to compute a risk score in the training (GSE65904) and validation cohorts (TCGA_SKCM, GSE22153, and GSE54467), using the following formula: –0.08209 × PSMB8 – 0.02401 × PLAAT4 – 0.18873 × GZMA – 0.19433 × GCH1. The cTMGs was calculated using the risk score of the patient subtracted by the minimum risk score of each cohort, which was then divided by the maximum risk score of the cohort, as reported in our previous studies (30, 31). CM patients in all these four datasets were divided into two groups using their respective median cTMGs as the cutoff value. The concordance of cTMGs-based stratification with the cTICscore-based division was evaluated; it was 70.33% in GSE65904, 78.95% in TCGA_SKCM, 76.47% in GSE22153, and 84.81% in GSE54467 (**Figure 3D**). The Kaplan–Meier survival analysis indicated that CM patients having low cTMGs had significantly better prognoses compared with those with high cTMGs (**Figure 3E**). Time-dependent ROC curves were used to calculate the AUC at different time points of the cTICscore and cTMGs. The result suggested that cTMGs had slightly improved predictive ability over the cTICscore for OS across the cohorts (**Figure 3F**).

### Relationship between cTMGs and clinical features of CM patients

**Figure 4:**
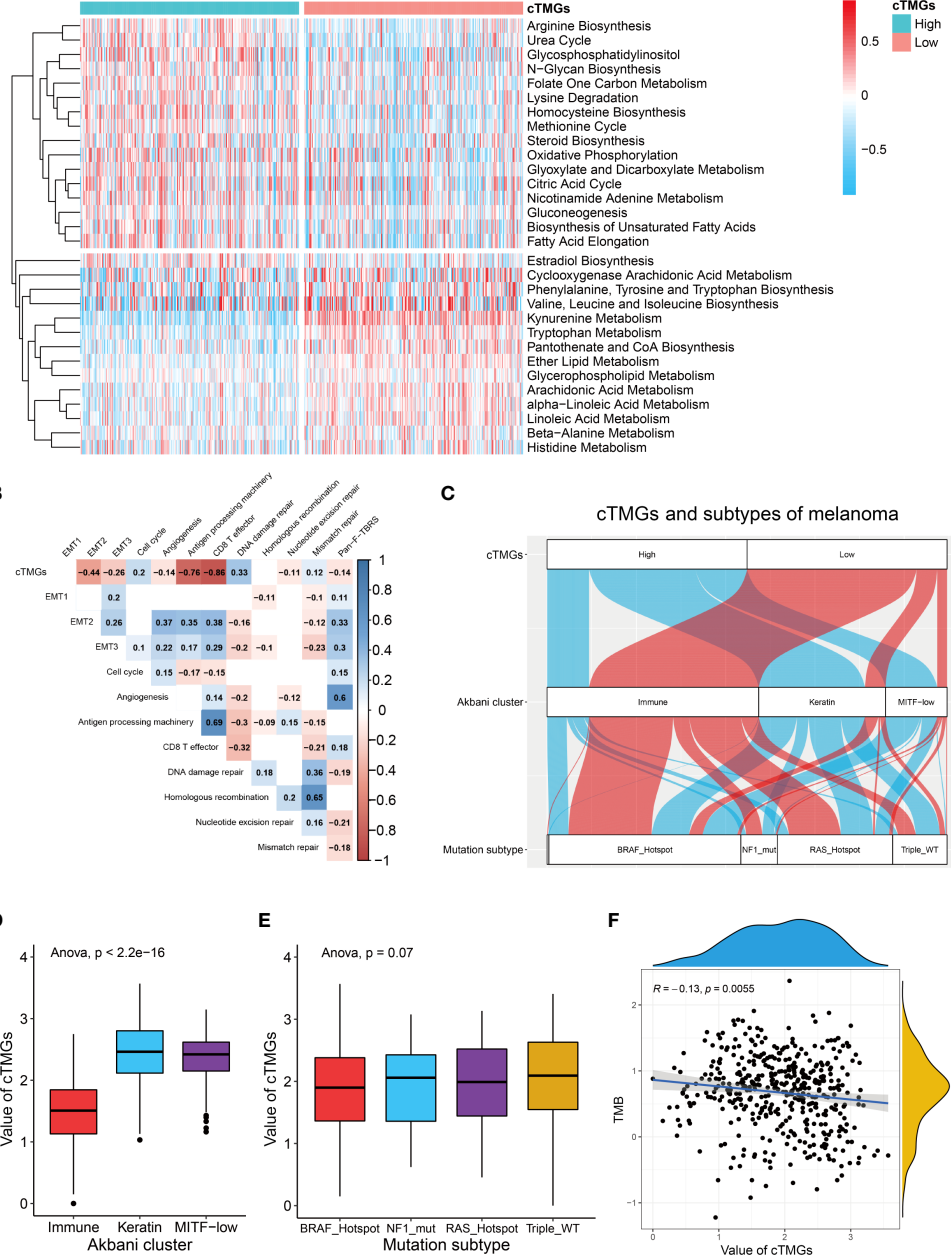
cTMGs negatively correlated with tumor immunity in CM. **(A)** Enrichment analysis of differential genes between high- and low-cTMGs groups. **(B)** Correlation analysis between cTMGs and the biological pathways in the TCGA_SKCM dataset. **(C)** Sankey diagram showed the connection degree between cTMGs, Akbani cluster, and mutation subtype in the TCGA_SKCM dataset. **(D)** Box plot showing a difference in the value of cTMGs across the three Akbani clusters for CM patients in the TCGA_SKCM dataset. **(E)** Box plot showing the difference in the value of cTMGs across the four mutation subtypes for CM patients in the TCGA_SKCM dataset. **(F)** Scatter plot shows the correlation between TMB and cTMGs.

Previous analysis showed that CM patients having a high cTICscore were enriched in metabolism-related pathways (**Figure 1D**). Since the cTMGs closely correlated with the cTICscore, we further explored whether cTMGs-based subclasses of CM patients had different metabolic characteristics. We quantified 115 metabolic processes using a set of genes identified by Chen Yang et al. (27). We found that the high-cTMGs subclass was predominantly enriched in energy metabolism–, lipid metabolism–, and glycan metabolism–related terms such as citric acid cycle, steroid biosynthesis, and gluconeogenesis (**Figure 4A**), while the low-cTMGs subclass was prone to be enriched in amino acid metabolism–related terms such as valine, leucine, and isoleucine biosynthesis, and kynurenine metabolism (**Figure 4A**).

We also evaluated the characteristics of specific biological activities of cTMGs-based subtypes using a set of genes identified by Mariathasan et al. (22). The antigen presentation machinery and CD8 effector signatures were significantly highly expressed in the low-cTMGs subtype of CM patients (**Figures S4A–C**). Consistently, the cTMGs showed a strong negative correlation with the antigen presentation machinery and CD8^+^T effector signatures (**Figure 4B**; **Figures S4D, E**). Although some epithelial to mesenchymal transition (EMT) markers, including EMT2, EMT3, and angiogenesis, were found to exhibit a significantly negative correlation with the cTMGs in the TCGA_SKCM dataset (**Figure 4B**), such a correlation could not be repeated in other CM cohorts (**Figures S4D, E**). Meanwhile, the enrichment score of most DNA damage repair–related signatures showed weak or no correlation with the cTMGs (**Figure 4B**; **Figures S4D, E**). CM patients were divided into “immune,” “keratin,” and “MITF-low” clusters based on the consensus hierarchical clustering analysis of the selected 1,500 genes (44). Our study found that CM patients in the low-cTMGs subgroup were predominantly distributed in the “immune” cluster, whereas the remaining patients were largely in the “keratin” and “MITF-low” clusters (**Figure 4C**). Consistently, the cTMGs was the lowest in the immune subtype (**Figure 4D**). CM patients were also divided into four subtypes based on the pattern of the most prevalent significantly mutated genes: mutant *B-Raf Proto-Oncogene*, *Serine/Threonine Kinase (BRAF)*, mutant *RAS*, mutant *Neurofibromin 1* (*NF1)*, and Triple-WT (wild-type) (44). However, no difference in the cTMGs was observed among these four subtypes (**Figure 4E**); the result was consistent with the lack of correlation between cTMGs and most DNA damage repair–related signatures (**Figure 4B**). Correspondingly, TMB showed a weak correlation with cTMGs (*r* = –0.13, *P* = 0.0055, **Figure 4F**).

### Immune landscape of CM patients classified by cTMGs

**Figure 5:**
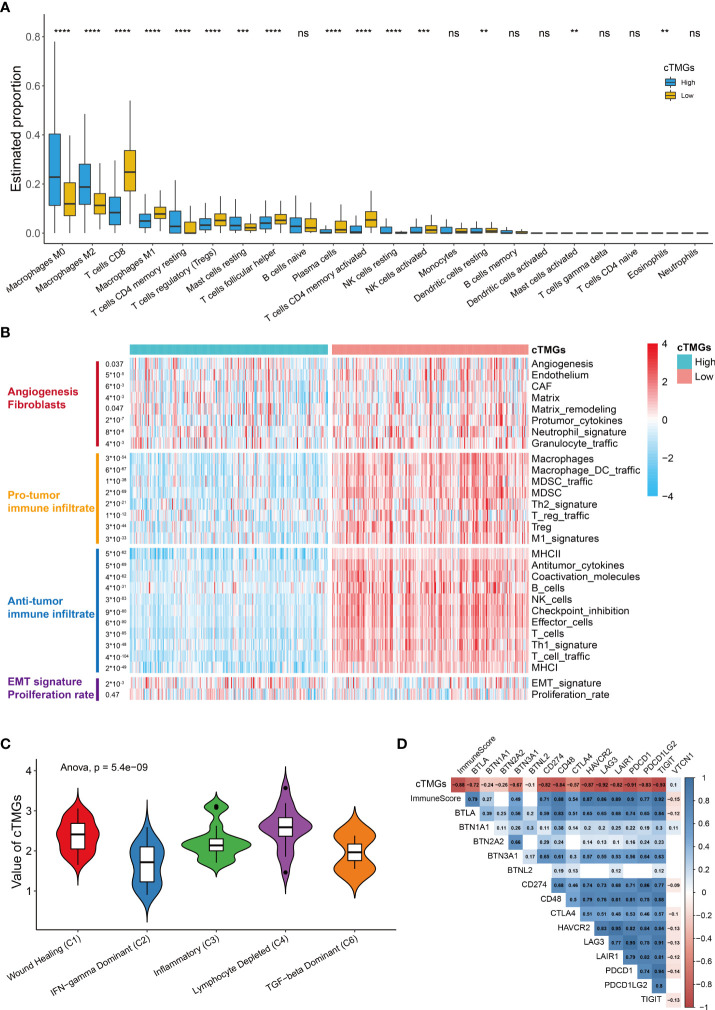
Immune infiltration in high- and low-cTMGs CM. **(A)** Distribution of infiltrating immune cells in high- and low-cTMGs groups CM. **(B)** Heatmap showed the 29 Fges in CM patients in the TCGA_SKCM dataset. **(C)** Box plot showing a difference in the value of cTMGs across the five subtypes for CM patients in the TCGA_SKCM dataset. **(D)** Heatmap shows the correlation between cTMGs and the expression levels of immune checkpoints *p < 0.05; **p < 0.01; ***p < 0.001; ****p < 0.0001. ns for no significance.

We further investigated the distribution of infiltrating immune cells in the low- and high-cTMGs groups of CM patients. We found that patients in the high-cTMGs group demonstrated significantly higher numbers of M0 and M1 macrophages, resting memory CD4^+^ T cells, and resting mast cells, whereas those in the low-cTMG group had a significantly higher proportion of CD8^+^T cells, M1 macrophages, regulatory T cells, follicular helper T cells, activated memory CD4^+^T cells, and activated NK cells (**Figure 5A**). In addition, CM patients in the high-cTMGs group presented an M2 phenotype, since the ratio of M2 macrophage/(M2 macrophage + M1 macrophage) was significantly higher in these patients (*P*< 2.2e-16, **Figure S5A**).

A recent study proposed a holistic characterization of TME by 29 functional gene expression signatures (Fges), which included the stromal compartments such as angiogenesis, immune suppression *via* macrophages and myeloid-derived suppressor cells, antitumor immune activities such as antigen processing, and infiltration of cytotoxic immune cells and Fges related to cancer cell properties (8). Further, four conserved subtypes were identified across 20 different cancers based on the following signatures: “Immune-Enriched, Fibrotic” (IE/F), “Immune-Enriched, Non-Fibrotic” (IE), “Fibrotic” (F), and “Depleted” (D) subtypes (8). In our study, we found that low-cTMGs CM was characterized by high levels of immune infiltrate and elevated expression of Fges associated with angiogenesis, matrix remodeling, and cancer associate fibroblast (CAF) activation (**Figure 5B**). Besides, EMT transition Fges was significantly upregulated in patients with high-cTMGs, whereas no difference in the proliferation rate was observed between the high- and low-cTMGs groups (**Figure 5B**). Indeed, 91.07% of CM patients in the high-cTMGs subgroup presented in the F and D subtypes, reflecting that the high-cTMGs subgroup had minimal or completely lacked leukocyte/lymphocyte infiltration (**Figure S5B**) (8). Correspondingly, the D subtype of CM patients had the highest cTMGs, followed by the F subtype. In addition, the IE/F and IE subtypes had the lowest level of cTMGs, reflecting the feature of this subtype exhibiting the most immune-active microenvironment among the four subtypes (**Figure S5C**) (8). Similar results were observed in the other two cohorts (**Figures S5D, E**).

Cancer patients were also divided into six subtypes in TCGA cohort (45). Our results demonstrated that the C2 subtype (IFN-γ dominant) had the lowest cTMGs, while the C4 samples (lymphocyte depleted) had the highest cTMGs (**Figure 5C**). Besides, we also found a strong negative correlation between cTMGs and Immunescore or the expression of most immune checkpoint molecules such as CD274, LAG3, PDCD1, and T-cell immunoreceptor with Ig and ITIM domains (TIGIT) (**Figure 5D**).

### cTMGs was shown to be predictive for the efficacy of immunotherapy

**Figure 6:**
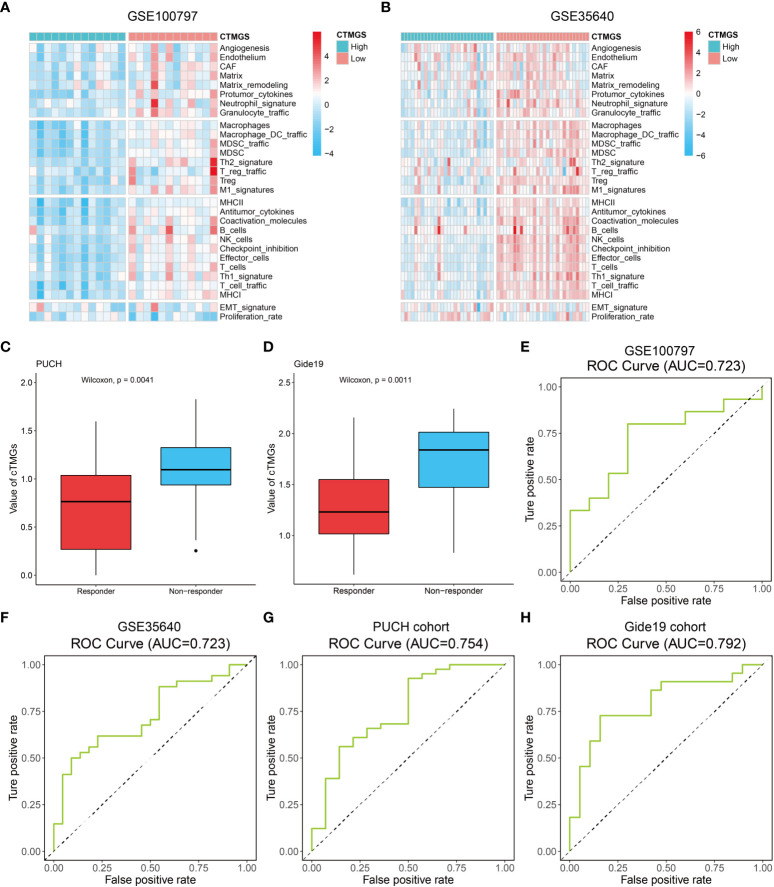
Performance of the cTMGs for predicting the effect of ICI therapy on CM patients. **(A, B)** Enrichment analysis of the 29 Fges of CM patients from the GSE100797 **(A)** and GSE35640 **(B)** dataset. **(C, D)** cTMGs value of CM patients receiving anti-PD-1 monotherapy in the PUCH cohort **(C)** or Gide19 cohort **(D)**. **(E–H)** ROC curve showing the performance of the cTMGs for predicting the effect of immunotherapy on CM patients in the GSE100797 **(E)**, GSE35640 **(F)** dataset, PUCH cohort **(G)**, or Gide19 cohort **(H)**.

The aforementioned analysis revealed that patients with low-cTMGs were enriched in both pro- and antitumor immune infiltrates and the cTMGs had a strong negative correlation with the expression of most targets of immune checkpoint inhibitors (ICIs). We hypothesized that treatments targeting these immune checkpoint molecules or pro-tumor immune infiltrates, or activating the function of antitumor immune cells, might lead to tumor shrinkage and improved prognosis. Four melanoma-related cohorts were analyzed in this work (GSE100797, GSE35640, PUCH cohort, and Gide19 cohort). Consistent with the previous result (**Figure 5B**), melanoma patients in the low-cTMGs group from GSE100797 and GSE35640 datasets also had high levels of pro- and antitumor immune infiltrates (**Figures 6A, B**). Moreover, melanoma patients who responded to the ICI therapy had a significantly lower cTMGs compared with non-responders (**Figures 6C, D**). Patients who showed complete response (CR) after adoptive T-cell therapy (ACT) treatment had the lowest cTMGs, whereas those who progressed had the highest cTMGs (CR vs. PD, *P* = 0.038, **Figure S6A**). Consistently, the level of tumor shrinkage significantly positively correlated with cTMGs (*P*< 0.05, *r* = 0.41, **Figure S6B**). In these four cohorts, a higher ratio of CM patients in the low-cTMGs group responded to immunotherapies (**Table 1**), and the AUC values of cTMGs in predicting response to these therapies were all great than 0.7 (**Figures 6E–H**). After immunotherapies, CM patients in the high-cTMGs subgroup tended to show a shorter progression-free survival (PFS, **Figure S6C**) or OS (*P* = 0.0055, **Figure S6D**) compared with those in the low-cTMGs group.

**Table 1:** Response to immunotherapies of cTMGs-stratified groups.

Dataset		cTMG-high	cTMG-low	p value
GSE100797	Responder	3	7	0.0722
	Non-responder	10	5	
GSE35640	Responder	7	15	0.0286
	Non-responder	21	13	
PUCH cohort	Responder	3	11	0.0165
	Non-responder	24	17	
Gide19 cohort	Responder	5	14	0.003
	Non-responder	16	6	
Kim18 cohort	Responder	3	10	0.0165
	Non-responder	20	12	

**Figure 7:**
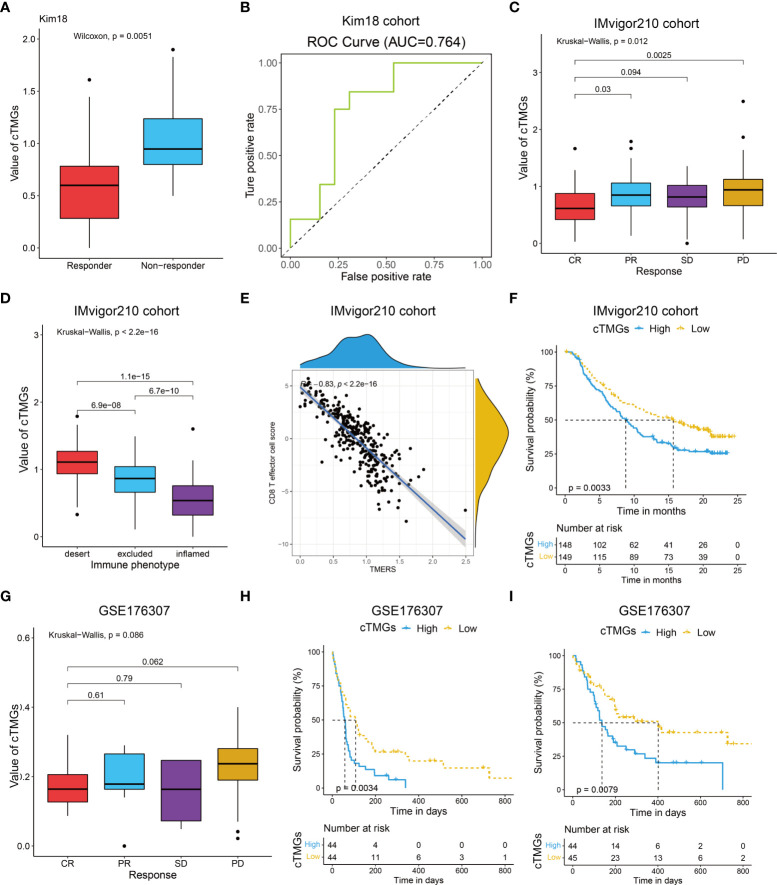
Performance of the cTMGs for predicting the effect of ICI therapy on non-CM patients. **(A)** Box plot showing a difference in the value of cTMGs across the two response subtypes for patients with gastric cancer in the Kim18 cohort. **(B)** ROC curve showing the performance of the cTMGs for predicting the effect of pembrolizumab therapy on patients with gastric cancer in the Kim18 cohort. **(C)** Box plot showing differences in the value of cTMGs across the four response subtypes of the IMvigor210 study. **(D)** Box plot showing differences in the value of the cTMGs across the three immune phenotype subtypes of the IMvigor210 study. **(E)** Scatter plot showing the correlation between CD8 T effector cell score and cTMGs in the IMvigor210 study. **(F)** OS of patients with UC in the IMvigor210 study stratified by cTMGs subtype classification. **(G)** Box plot showing differences in the value of cTMGs across the four response subtypes for the GSE176307 dataset. **(H, I)** OS **(H)** and PFS **(I)** of patients with UC in the GSE176307 dataset stratified by cTMGs subtype classification.

We also evaluated the value of the cTMGs in non-CM cohorts. In the Kim18 cohort in which 55 patients with gastric cancer were treated with pembrolizumab (46), a significantly lower level of the cTMGs was observed in those who responded to the therapy (*P* = 0.0051, **Figure 7A**). Consistently, the ORR in the low-cTMGs subgroup of patients with gastric cancer was higher than that in the high-cTMGs subgroup (45.45% vs. 13.04%, *P* = 0.0165, **Table 1**). In this cohort, an AUC of 0.764 was achieved (**Figure 7B**). Two cohorts of patients with metastatic urothelial cancer (UC) receiving ICI were also investigated. As shown in **Figures 7C, G**, patients with UC who progressed from ICI treatment had higher cTMGs compared with those who showed CR after immune therapy. Patients with UC classified in the inflamed subgroup, or with a high PD-L1 expression on either tumor cells (TC2+) or immune cells (IC2+), showed the lowest cTMGs (**Figure 7D**; **Figure S6E, F**). In addition, cTMGs had a strong negative correlation with the CD8^+^T effector cell score (*P*< 2.2e-16, *r* = –0.83, **Figure 7E**). Kaplan–Meier analyses demonstrated that patients with UC in the low-cTMGs group could enjoy survival benefit from immunotherapy compared with those in the high-cTMGs group (**Figures 7F, H, I**).

## Discussion

The TME is not only a major factor affecting CM progression but also a promising target of tumor therapy (47). Accumulated evidence indicated that the fraction of immune cells in the TME could serve as a marker for the diagnosis and prognosis of a variety of malignant tumors (48, 49). However, some prognostic models based on the TME were developed in the setting of a pan-cancer analysis (50, 51); they might not be the most optimal choice for CM. In addition, not all fractions of the TME exert a significant impact on the prognosis of tumors. Based on the aforementioned consideration, we identified core TME components that had a close association with the prognosis of CM patients and proposed a new index (cTICscore). Although the cTICscore has a strong predictive capability for the CM patients’ prognosis, the acquisition process for a fraction of the four core immune cells is cumbersome.

Previous studies showed that CD8^+^T cells played a central role in mediating antitumor immunity. CD8^+^T cells can release perforin and IFN-γ to kill melanoma cells (13). Besides, CD4^+^T cells play an antitumor role by secreting IFNγ and IL-4, recruiting effector cells including eosinophils and macrophages and helping CD8^+^T cells (52). NK cells play a vital role in initiating antitumor response, but resting NK cells harbor poor effector function (53). The decreased cytotoxicity of resting NK cells may be due to the decreased expression of granzyme B and perforin (54). M1 macrophages can also play an antitumor role by producing pro-inflammatory molecules and presenting tumor-specific antigens to T cells (55). As we mentioned above, the four core TME components, which have an impact on the prognosis of CM patients, were found out to be associated with certain genes related to cTICscore we have screened. The expressive level of these genes influences the release of these components and therefore affects the TME and tumor development.

Based on the pivotal role the four key genes have played, we generated the cTMGs, consistent with the cTICscore, to be a more suitable and comprehensive marker over the cTICscore for certain advantages in prognosis prediction and clinical use. “Deregulating Cellular Energetics” is a hallmark of cancer (56); obviously, the rapid proliferation of malignant tumor cells requires a lot of energy (57). The divided groups of patients by cTMGs present distinct characteristics. While the low-cTMGs group was highly enriched in the amino acid metabolism-related gene sets and immune subtype reflecting a hot immune feature, the high-cTMGs group was found to be predominantly enriched in energy metabolism–related gene sets (27), consistent with the fact that dysregulation of energy might contribute to the rapid proliferation of tumor cells and the results of Kaplan–Meier survival analysis. Part of the contribution to the rapid proliferation may be due to the impact the upregulation of specific metabolism-related pathways has on the TME of the high-cTMGs group. For instance, the downregulation of the activity of pyruvate kinase, a component of glucose metabolism, is associated with increased aerobic glycolysis (58), facilitating cancer cell proliferation and tumor enlargement. It also reflects the increase in lipogenesis, fatty acid (FA) uptake, and FA oxidation owing to the production of plasma membrane synthesis and energy requirement of the expanded tumor cells (59).

TMB, a leading potential biomarker for identifying cancer patients benefiting from immunotherapies, measures the number of somatic mutations per megabase (Mb) of the interrogated genomic sequence of a tumor (60). Theoretically, the increase in the number of mutant proteins will create antigenic peptides allowing for enhanced immunogenicity (61). However, retrospective analyses of a bunch of cancer patient cohorts suggested that high TMB, compared to low TMB, fails to indicate an improved response rate to ICIs for certain cancer types, and neoantigen load does not always show a significant correlation with CD8 T-cell infiltration (61). These results suggest that TMB does not always show a clear cause-and-effect relationship with the infiltration of immune cells into tumors, and the components of the TME could be affected by numerous non-TMB factors such as hypoxia conditions. The complexity of the TME might help to explain why cTMGs, a reflection of the level of intra-tumor immune cells, was weakly correlated with TMB and DNA damage repair–related signatures.

Furthermore, cTMGs showed a strong negative correlation with the antigen presentation machinery and CD8^+^T effector signatures. Tumor immunogenicity is mainly determined by tumor antigenicity and antigen presentation efficiency (62). Chowell et al. and Zaretsky et al. reported that antigen presentation defects contributed to ICI response failure (63, 64). The grouping pattern of CM patients according to cTMGs was further supported by studies of “Cancer Genome Atlas Network” and Bagaev et al. (8, 44). In addition, cTMGs strongly negatively correlated with the expression of most ICIs. Based on these findings, we presumed that patients with a high cTMGs might have a poor response to immunotherapy.

To confirm our conjecture, we compared the immune response of cTMGs-based subgroups from four independent CM cohorts. We found that CM patients in the low-cTMGs group had a significantly higher response to ACT, MAGE-A3 antigen-specific cancer immunotherapy, anti-PD-1 monotherapy, or anti-PD-1/anti-CTLA-4 combined therapy compared with those in the high-cTMGs group.

Consequently, the abovementioned presumptions have a great potential to be applied in clinical use. After collecting the gene-expression information of the patient, cTMGs can be calculated subsequently to reveal the general landscape of his or her TME condition and possible outcome. Based on these materials, a more prognosis-oriented immunotherapy can be constructed for a more responsive and accurate treatment.

Similar conclusions in patients with gastric cancer and metastatic urothelial carcinoma were obtained, suggesting that cTMGs not only served as a prognostic factor for CM immunotherapy but also had the potential to be applied in other tumors. Although immunotherapy has changed the treatment landscape of many tumors, how exactly “cold tumors” benefit from ICIs remains a big challenge (65). The patients in the high-cTMGs group had a low fraction of T-cell infiltration and dysregulated energy-related pathways, which were consistent with the characteristics of “cold tumors.” Boosting T-cell infiltration into the TME is essential for ameliorating the immunotherapeutic effect (66, 67). Theoretically, NK cell–based approaches, oncolytic viruses, pattern recognition receptor (PRR) agonists, CD 40 agonistic antibodies, transforming growth factor beta (TGF-β)-blocking antibodies and TGF-β-receptor antagonists, immunocytokines, and T-cell-recruiting bispecific antibodies might overcome the absence of T-cell infiltration in tumors, including CM patients in the high-cTMGs group (65, 66). Approaches influencing energy metabolism are also potential directions. More studies are still required to bring hope from bench to bedside.

In conclusion, we identified the core components in the TME of CM which helped us understand their importance for immunotherapy. The cTMGs can be used to stratify CM patients with distinct prognosis and identify those who can benefit more from immunotherapy.

## Data availability statement

The datasets presented in this study can be found in online repositories. The names of the repository/repositories and accession number(s) can be found in the article/**Supplementary Material**.

## Author contributions

YH and ZS designed and directed the study. ZZ, GL, and ZL conducted the data analysis and data interpretation, YW, YY, MW, HZ, and HQ conducted the data collection. ZZ and GL wrote the manuscript. All authors have read and verified the underlying data and approved the final version of the manuscript.

## Funding

This work was supported by the Youth Program of the National Natural Science Foundation of China (No. 81903031); the open project of the Key Laboratory of Modern Teaching Technology, Ministry of Education (No. SYSK202107); and the Luzhou Municipal People’s Government-Southwest Medical University Science and Technology strategic cooperation project (No. 2021LZXNYD-J25). The funders had no role in study design, data collection, interpretation and analysis, decision to publish, or preparation of the manuscript.

## Conflict of interest

The authors declare that the research was conducted in the absence of any commercial or financial relationships that could be construed as a potential conflict of interest.

The reviewer YW declared a shared affiliation with the author ZL to the handling editor at time of review.

## Publisher’s note

All claims expressed in this article are solely those of the authors and do not necessarily represent those of their affiliated organizations, or those of the publisher, the editors and the reviewers. Any product that may be evaluated in this article, or claim that may be made by its manufacturer, is not guaranteed or endorsed by the publisher.
