# Supplementary material for: Core immune cell infiltration signatures identify molecular subtypes and promote precise checkpoint immunotherapy in cutaneous melanoma
Source: Front Immunol. 2022 Aug 22;13:914612. doi: 10.3389/fimmu.2022.914612 (PMC9441634; doi:10.3389/fimmu.2022.914612)
Supplement: Supplementary file 1 [file DataSheet_1.doc]

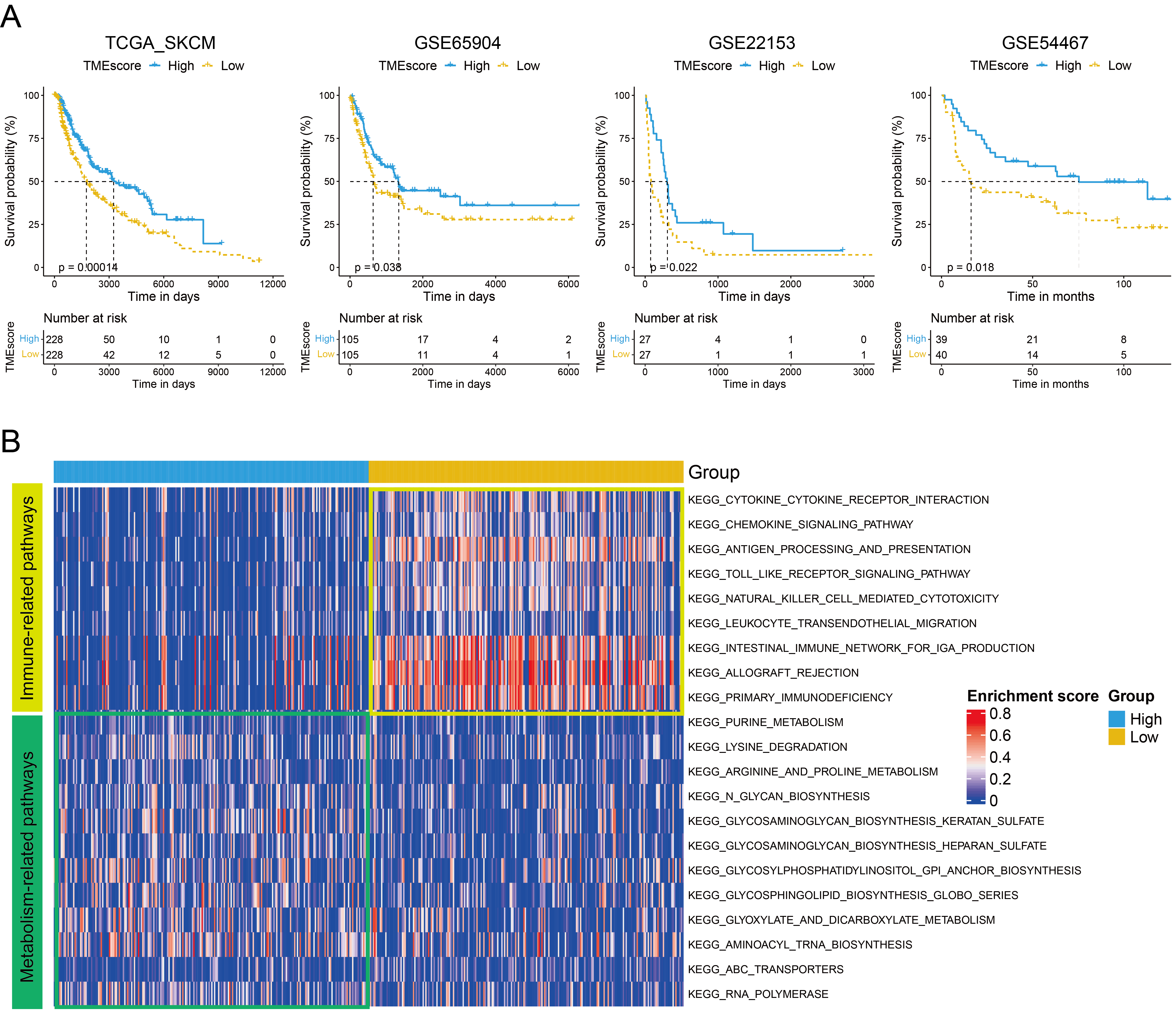


**Supplementary Figure 1. Survival analysis and signal pathway enrichment analysis of CM patients**
(A) OS of CM patients in TCGA_SKCM, GSE65904, GSE22153 and GSE54467 datasets stratified by TMEscore subtype classification. (B) KEGG pathways enriched in the high- and low- cTICscore groups CM patients in TCGA_SKCM dataset.


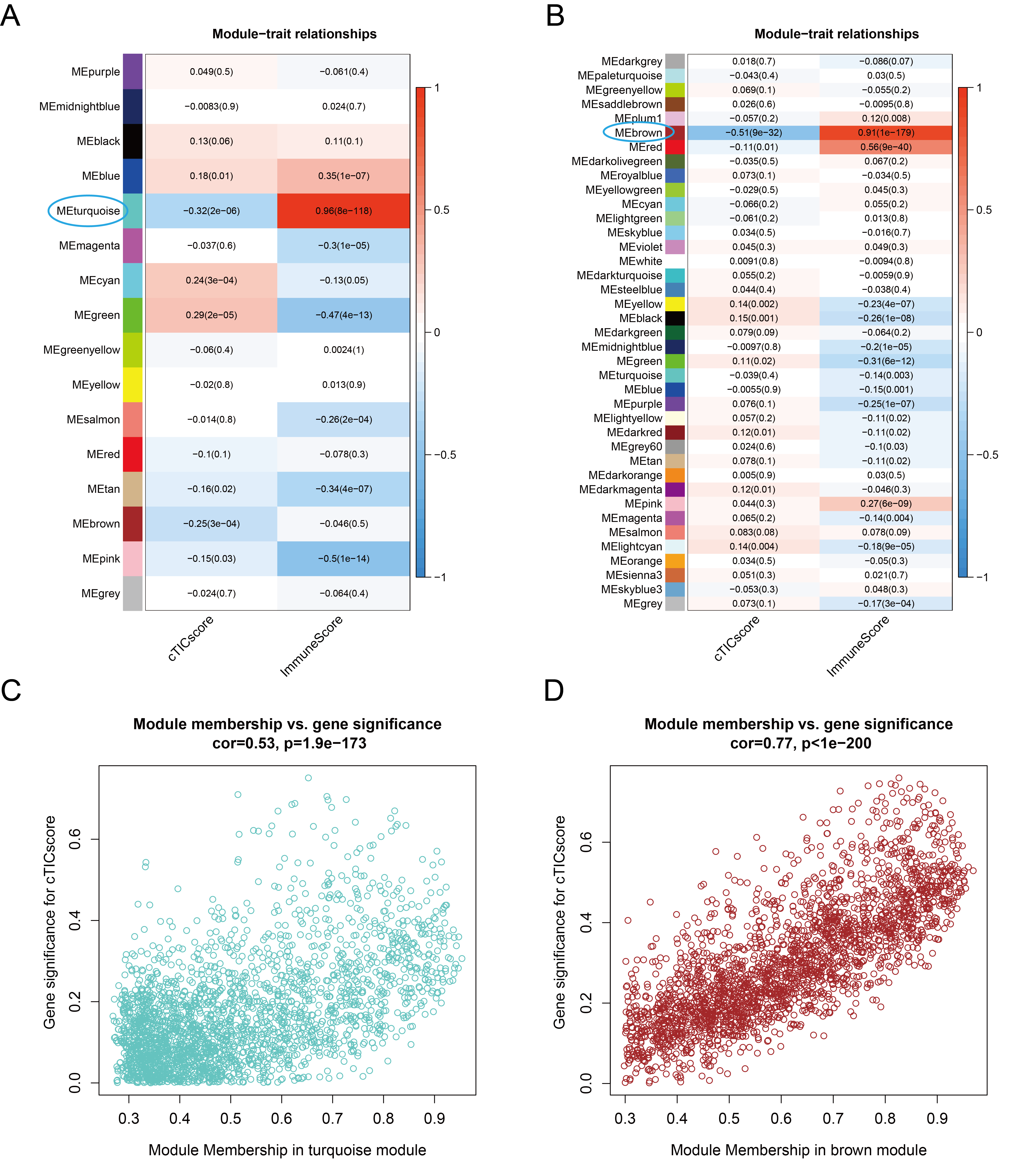


**Supplementary Figure 2. Identification of module genes highly correlated with cTICscore**(A&B) Correlation heatmaps of module genes and cTICscore or Immunescore in GSE65904 (A) and TCGA_SKCM (B) datasets. The correlation coefficient changed from -1 to 1 as the color turned from blue to red gradually. (C) Scatter plot showing correlation between gene significance for cTICscore and the turquoise module genes in the GSE65904 dataset. (D) Scatter plot showing correlation between gene significance for cTICscore and the brown module genes in the TCGA_SKCM dataset.


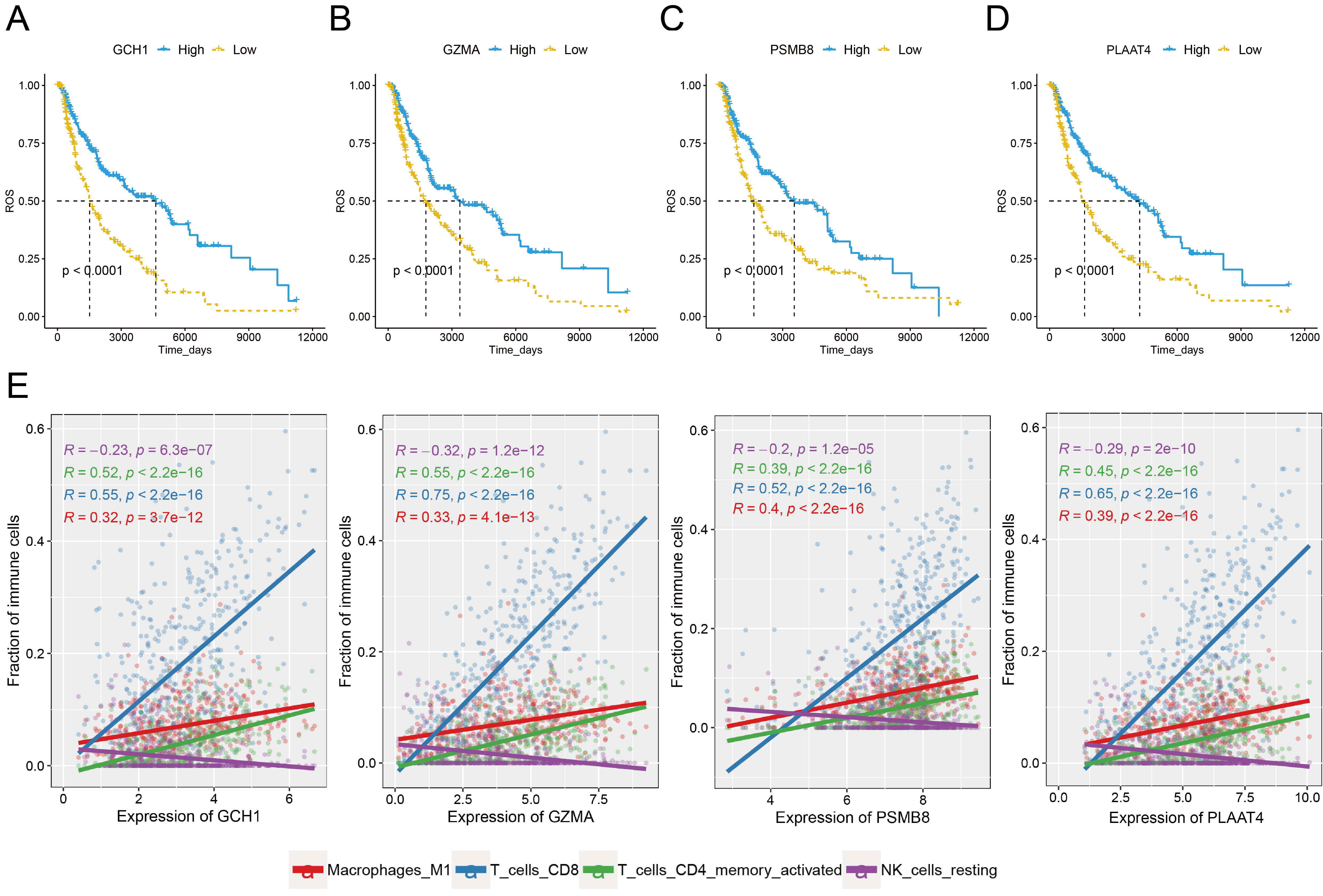


**Supplementary Figure 3. Correlation analysis of the four crucial genes with tumor immune infiltration and prognosis**

1. D) OS of CM patients in TCGA_SKCM dataset stratified by the media value of the four crucial genes. (E) Scatter plot showing correlation between the expression levels of four crucial genes and the fraction of immune cells.


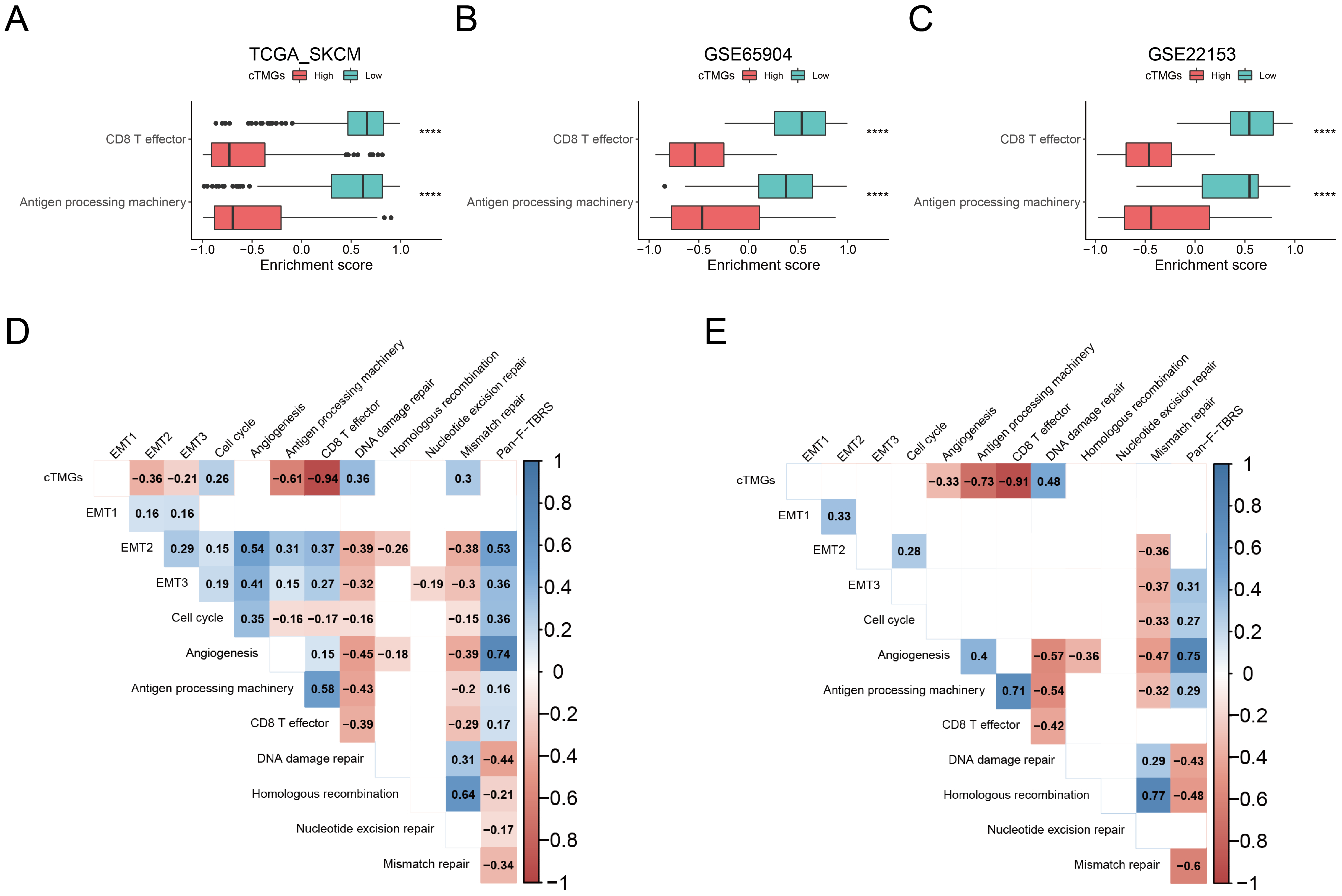


**Supplementary Figure 4. Correlation between cTMGs and biological pathways in CM**(A-C) Box plot showing difference in the antigen presentation machinery and CD8 effector signatures across the two cTMGs subtypes for CM patients in TCGA_SKCM, GSE69504, and GSE22153 datasets. (D&E) Correlation analysis between cTMGs and the biological pathways in GSE69504 (D) and GSE22153 (E) datasets.


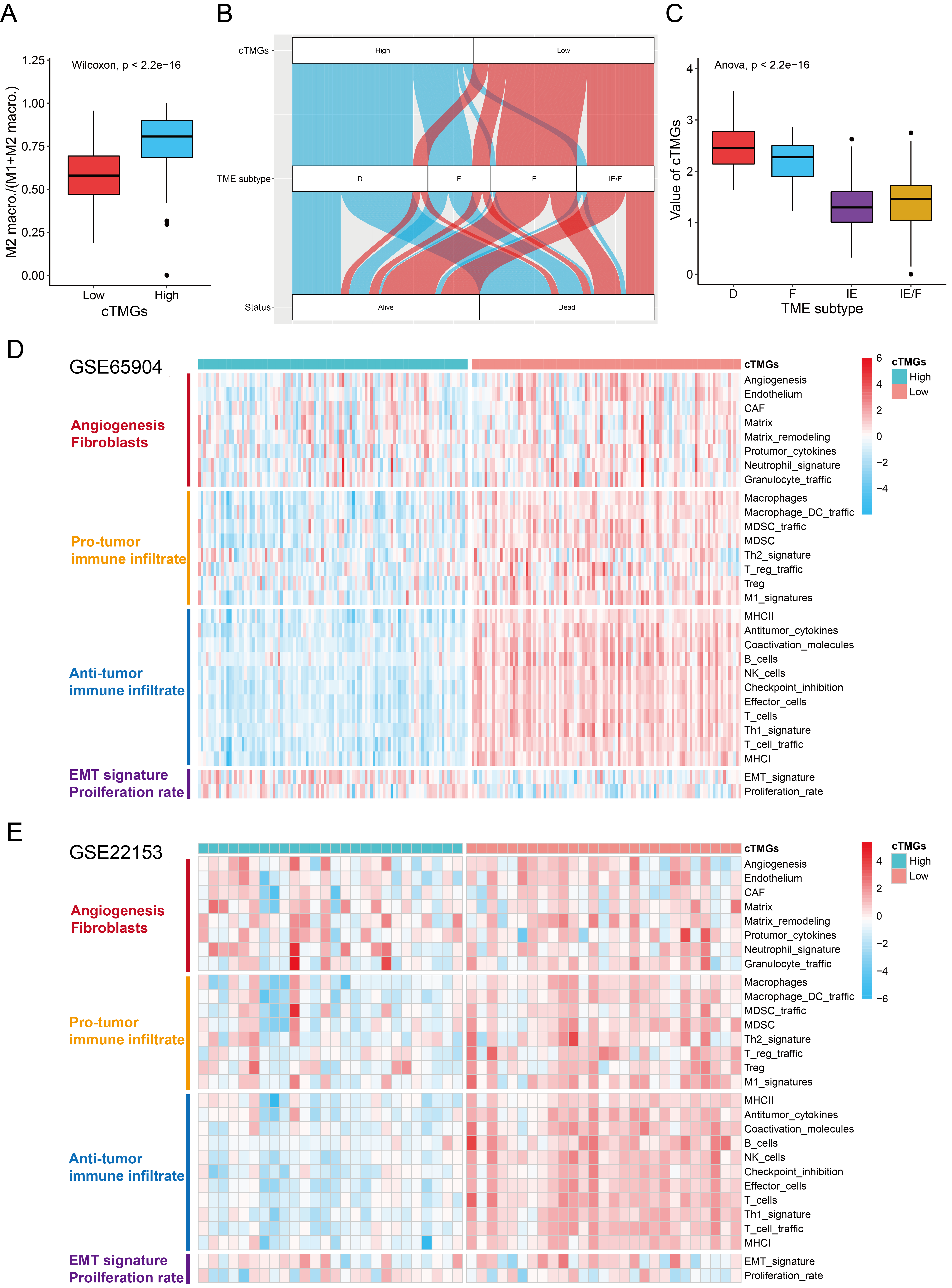

**Supplementary Figure 5. The immune landscape of CM patients classified by cTMGs**(A) Box plot showing difference in the ratio of M2 macrophage / (M2 macrophage + M1 macrophage) across the two cTMGs subtypes for CM patients in TCGA_SKCM dataset. (B) Sankey diagram showed the connection degree between cTMGs, TME subtype, and statu in the TCGA_SKCM dataset. (C) Box plot showing difference in the value of cTMGs across the four TME subtypes for CM patients in the TCGA_SKCM dataset. (D&E) Heatmap showed the 29 Fges in CM patients in the GSE65904 and GSE22153 datasets.


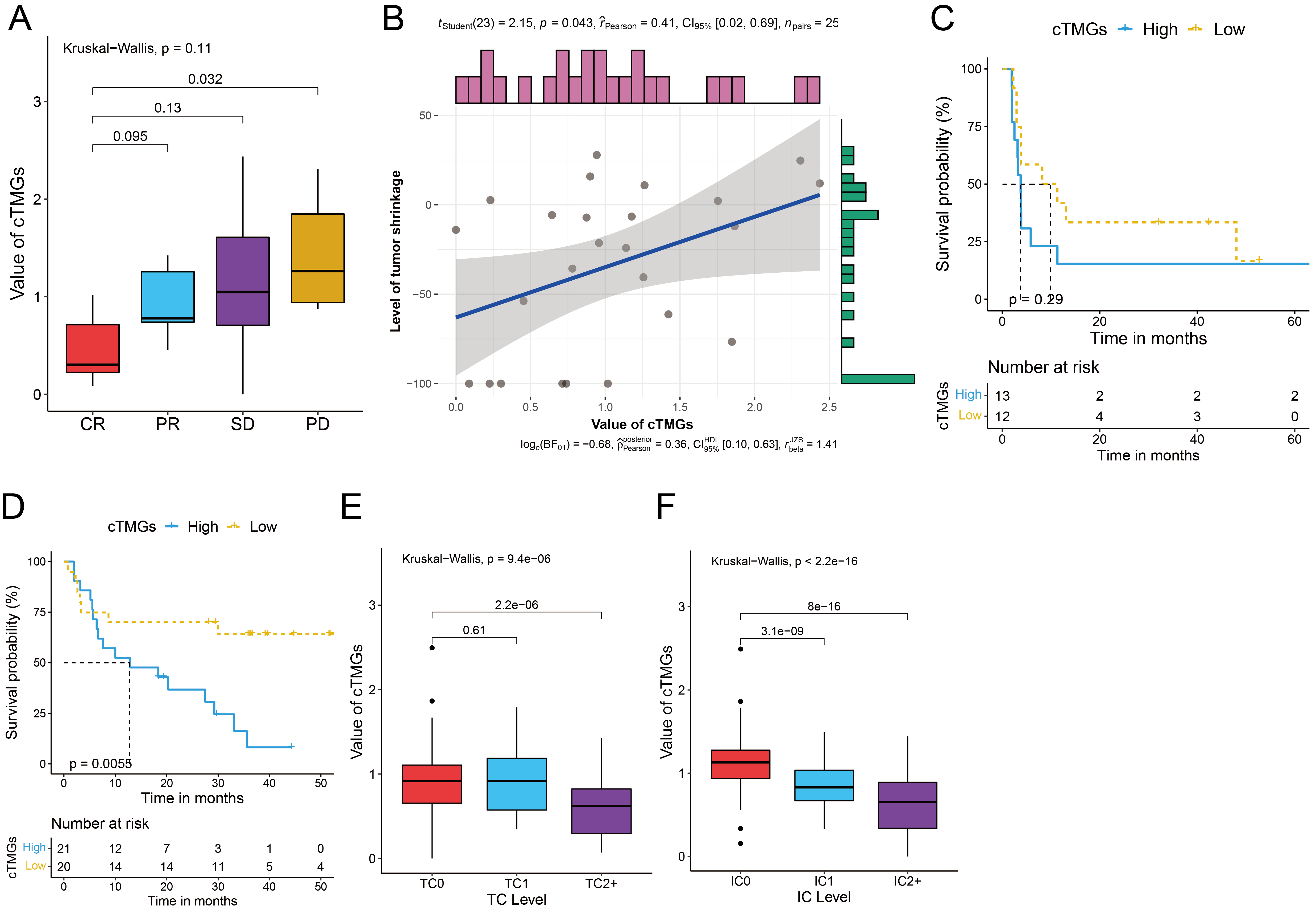


**Supplementary Figure 6.**(A)Box plot showing difference in the value of cTMGs across the four subtypes for CM patients in the TCGA_SKCM dataset. (B) Scatter plot showing correlation between the level of tumor shrinkage and cTMGs in the TCGA_SKCM dataset. (C) PFS of 25 CM patients receiving adoptive T-cell therapy stratified by cTMGs subtype classification. (D) OS of 41 CM patients in Gide19 cohort stratified by cTMGs subtype classification. (E&F) Box plot showing difference in the value of cTMGs across the three TC and IC subtypes for UC patients in the IMvigor210 study.

**Supplementary Table1** The fraction of 22 immune cells was estimated using the CIBERSORT algorithm in three independent CM-related datasets.

| **celltype** | **TCGA_SKCM** |  | **GSE65904** |  | **GSE22153** |  |
| --- | --- | --- | --- | --- | --- | --- |
|  | **HR (95% CI for HR)** | ***p*.value** | **HR (95% CI for HR)** | ***p*.value** | **HR (95% CI for HR)** | ***p*.value** |
| Neutrophils | 7.1 (0.0093-5500) | 0.56 | 19 (0.0045-81000) | 0.49 | 3.2e+21 (4.4e+09-2.4e+33) | 0.00038 |
| NK_cells_resting | 89 (1.8-4500) | 0.024 | 8100000 (110-5.8e+11) | 0.0053 | 740000 (250-2.2e+09) | 0.00091 |
| Mast_cells_activated | 11 (0.013-9900) | 0.48 | 2500 (2.1e-09-2.9e+15) | 0.58 | 2.3e+11 (20000-2.5e+18) | 0.0016 |
| T_cells_follicular_helper | 0.032 (0.00032-3.3) | 0.15 | 0.56 (0.0023-140) | 0.84 | 1.9e-05 (3.9e-09-0.09) | 0.012 |
| Macrophages_M1 | 0.0045 (0.00017-0.12) | 0.0013 | 0.026 (0.00024-2.8) | 0.13 | 0.00014 (9.6e-08-0.19) | 0.016 |
| Monocytes | 14 (0.16-1100) | 0.25 | 36 (0.8-1600) | 0.065 | 170 (0.8-38000) | 0.06 |
| Macrophages_M0 | 2.7 (1.3-5.6) | 0.0059 | 3.6 (0.9-15) | 0.07 | 6.5 (0.83-51) | 0.075 |
| T_cells_gamma_delta | 0.0041 (1.8e-06-9.6) | 0.16 | 0.00041 (3e-06-0.055) | 0.0018 | 0.0079 (1.2e-05-5.2) | 0.14 |
| T_cells_regulatory_Tregs | 4.5 (0.14-140) | 0.39 | 680 (0.0028-1.6e+08) | 0.3 | 0.00044 (4.4e-09-44) | 0.19 |
| B_cells_memory | 0.31 (0.0019-53) | 0.66 | 0.41 (0.0021-78) | 0.74 | 0.039 (0.00014-11) | 0.26 |
| T_cells_CD4_memory_resting | 2.3 (0.24-22) | 0.47 | 12 (0.8-180) | 0.073 | 11 (0.16-820) | 0.27 |
| Plasma_cells | 0.38 (0.01-14) | 0.6 | 0.0052 (1.9e-05-1.4) | 0.066 | 0.061 (0.00039-9.7) | 0.28 |
| T_cells_CD8 | 0.15 (0.05-0.43) | 0.00048 | 0.016 (0.0013-0.19) | 0.0012 | 0.097 (0.00073-13) | 0.35 |
| NK_cells_activated | 8.6e-05 (5.2e-08-0.14) | 0.013 | 14 (0.029-6700) | 0.4 | 0.084 (1.6e-05-430) | 0.57 |
| Macrophages_M2 | 2.3 (0.67-7.8) | 0.19 | 2.4 (0.29-19) | 0.42 | 2.6 (0.082-82) | 0.59 |
| B_cells_naive | 1.6 (0.17-16) | 0.68 | 4.2 (0.0094-1900) | 0.64 | 4.7 (0.0097-2300) | 0.62 |
| T_cells_CD4_memory_activated | 0.0053 (0.00015-0.19) | 0.0041 | 0.0085 (0.00011-0.64) | 0.031 | 0.15 (6e-05-390) | 0.64 |
| Dendritic_cells_resting | 6.6 (0.014-3100) | 0.55 | 0.0012 (3.2e-08-45) | 0.21 | 40 (1.7e-06-9.2e+08) | 0.67 |
| Dendritic_cells_activated | 2.8e-07 (2.8e-18-28000) | 0.24 | 1600 (0.2-1.4e+07) | 0.11 | 0.0012 (3.6e-28-4e+21) | 0.82 |
| T_cells_CD4_naive | 210 (0.63-72000) | 0.071 | 0.44 (0.00013-1500) | 0.85 | 0.71 (0.0071-72) | 0.89 |
| Mast_cells_resting | 3.7 (0.19-76) | 0.39 | 57 (1.8-1900) | 0.022 | 0.86 (0.01-70) | 0.94 |
| Eosinophils | 2.8e+17 (0.0022-3.6e+37) | 0.089 | 1.1e-07 (1.6e-43-7.7e+28) | 0.7 | NA | NA |
